# Modelling of Cellular Survival Following Radiation-Induced DNA Double-Strand Breaks

**DOI:** 10.1038/s41598-018-34159-3

**Published:** 2018-11-01

**Authors:** Wenjing Wang, Chunyan Li, Rui Qiu, Yizheng Chen, Zhen Wu, Hui Zhang, Junli Li

**Affiliations:** 1 Department of Engineering Physics, Tsinghua University, Beijing, China; 2 Key Laboratory of Particle & Radiation Imaging (Tsinghua University), Ministry of Education, Beijing, China; 3 Nuctech Company Limited, Beijing, China

## Abstract

A mechanistic model of cellular survival following radiation-induced DNA double-strand breaks (DSBs) was proposed in this study. DSBs were assumed as the initial lesions in the DNA of the cell nucleus induced by ionizing radiation. The non-homologous end-joining (NHEJ) pathway was considered as the domain pathway of DSB repair in mammalian cells. The model was proposed to predict the relationship between radiation-induced DSBs in nucleus and probability of cell survival, which was quantitatively described by two input parameters and six fitting parameters. One input parameter was the average number of primary particles which caused DSB, the other input parameter was the average number of DSBs yielded by each primary particle that caused DSB. The fitting parameters were used to describe the biological characteristics of the irradiated cells. By determining the fitting parameters of the model with experimental data, the model is able to estimate surviving fractions for the same type of cells exposed to particles with different physical parameters. The model further revealed the mechanism of cell death induced by the DSB effect. Relative biological effectiveness (RBE) of charged particles at different survival could be calculated with the model, which would provide reference for clinical treatment.

## Introduction

Cellular response towards ionizing irradiation, especially cell death induced by ionizing irradiation has gained significant and wide interest since ionizing irradiation being used in cancer treatment. Clonogenic survival is an important endpoint to measure the cellular response towards ionizing irradiation. For many years, experimental studies on clonogenic cell killing attributed to ionizing irradiation have been conducted and published. Based on the experimental data, a number of models of clonogenic cell survival curve have been proposed to predict the relationship between energy deposition in cells and probability of cell survival.

The target theory was the initial exploration of the relationship between energy deposition in cells and probability of cell survival, which was of great significance for later theories. Based on the dual radiation action theory and molecular theory, the linear quadratic (LQ) model^1,2^ was proposed. The LQ model is the most frequently used method to quantitatively describe the response to ionizing irradiation, which dominates in clinical radiotherapy for many years^3^. In recent advances such as stereotactic radiotherapy (SRT), the target is delivered a few fractions of very large dose per fraction. For improving descriptions of high dose survival responses, different models have been proposed, such as the Padé Linear Quadratic (PLQ) model^4–6^, the Universal Survival Curve (USC) model^7^ and the Linear-Quadratic-Linear (LQL) model^8^, which were proved to be theoretically well-founded and useful in clinical applications at high doses as well as medium and low doses^9^. These models are phenomenological models, which are in good agreement with experimental data. However, the mechanistic drivers of radiation response have not been sufficiently revealed in these models. The model with fitting parameters obtained from experimental data only allows to calculate cell survival for specific radiation used in the experiment.

Advanced radiotherapy techniques which make use of protons and carbon ions have also been widely used recently^10,11^. As experimental studies only allow to calculate cell survival for specific irradiation conditions, a number of models have been proposed to predict cell survival for mixed beams with the concept of relative biological effectiveness (RBE) of protons and carbon ions to photons^12^. Besides the phenomenological mixed beam model^13^, mechanism models have been proposed, such as the local effect model (LEM)^14–16^, and the microdosimetric kinetic model (MKM)^17,18^. The mixed beam models are in good agreement with experimental data and have been clinically applied^19–22^. To predict the RBE of protons and carbon ions more accurately, some other approaches have been proposed as well^23–25^. The mechanistic drivers of radiation response have been discussed in the mechanism models. The models allow to predict the relationship between physical parameters of radiation and probability of cell survival.

To characterize cellular response towards ionizing irradiation at the molecular and cellular level, such as radiation-induced DNA damage, DNA damage repair, chromosome aberration formation and consequent cell death, several mechanistic models have been proposed too. The mechanistic models include the repair-misrepair-fixation (RMF) model proposed by Stewart *et al*.^26^, the biophysical analysis of cell death and chromosome aberrations (BIANCA) model proposed by Ballarini *et al*.^27–29^, the mechanistic modelling of DNA repair and cellular survival following radiation-induced DNA damage proposed by Stephen *et al*.^30,31^, etc.

DSBs induced by deposition of energy from the radiation in the DNA within the nucleus play a central part in chromosomal aberrations and cell killing attributed to radiation exposure at the molecular and cellular level^32^. To predict the relationship between radiation-induced DSBs in the nucleus and probability of cell survival, a mechanistic model of cellular survival following radiation-induced DSBs was proposed in this study. The proposed model has two input parameters and six fitting parameters. The input parameters of the model are the average number of primary particles which caused DSB and the average number of DSBs yielded by each primary particle that caused DSB. The fitting parameters are used to describe the biological characteristics of the irradiated cells. With the fitting parameters obtained from experimental data, the model allows to estimate surviving fractions for the same type of cell exposed to different particles at different linear energy transfer (LET).

## Methods

### Yield of radiation-induced DSBs

The best fits to the experimental data of dose response of radiation-induced DSBs were obtained by linear regression analysis. It indicated that for most of the radiation-induced DSBs, the two strand breaks (SBs) of each DSB were induced by the same primary particle. Therefore the average number of radiation-induced DSBs per cell, *N*, is given by:

1 $$N=Y\times D$$

where *Y* is the DSB yield per cell per Gy, and *D* (Gy) is the radiation dose to the nucleus.

For a cell nucleus with radius *R* (μm) and density *ρ* (g/cm^3^), the number of primary particle passing through the nucleus, *n*, can be derived by the LET (keV/μm):

2 $$n=\frac{\pi {R}^{2}\times D\times \rho }{LET\times 1.602\times {10}^{-19}}\times {10}^{-18}$$

Therefore, the DSB yield per cell per primary particle, *λ*, can be derived by:

3 $$\lambda =\frac{N}{n}$$

DSBs are assumed as the initial lesions in the DNA of the nucleus caused by ionizing radiation, therefore the primary particles which caused no DSB have made no contribution to cell death. By assuming that the number of DSBs yielded by a primary particle is Poisson-distributed, the probability of a primary particle passing through a cell nucleus without causing any DSB can be calculated with:

4 $${\rm{P}}(X=0)=\frac{{\lambda }^{0}}{0!}{e}^{-\lambda }={e}^{-\lambda }$$

The following equations can be derived then:

5 $${n}_{p}=n(1-{e}^{-\lambda })=\,\frac{YD}{\lambda }(1-{e}^{-\lambda })$$

6 $${\lambda }_{p}=\frac{\lambda }{1-{e}^{-\lambda }}$$

where *n*_*p*_ is the average number of primary particles that cause DSB, and *λ*_*p*_ is the average number of DSBs yielded by each primary particle that causes DSB.

In this study, the yield of DSBs induced by ionizing radiation was calculated with the fast Monte Carlo damage simulation (MCDS) software, which has been widely used to simulate DNA damage induced by ionizing radiation. The allowed particle types include electron, H-1, He-3, He-4, C-12, N-14, O-16, Ne-20 and Fe-56^33,34^. DSB yield per cell per Gy, *Y*, and DSB yield per cell per primary particle, *λ*, were directly obtained with MCDS. Then *n*_*p*_ could be calculated with equation (5), and *λ*_*p*_ could be calculated with equation (6).

### Repair of DSBs

In mammalian cells, the two most important types of DSB repair processes are the homologous recombination repair (HRR) pathway and the nonhomologous end-joining (NHEJ) pathway^12^. As the NHEJ pathway is the dominating pathway of DSB repair, and the probability that DSB being repaired correctly by HRR pathway is considerable high, only the NHEJ pathway was considered as having made contribution to cell death in the model. Each DSB has two DSB ends. Each DSB end may undergo one of four transformations: (a) it may be joined with the other end from the same DSB, (b) it may be joined with a DSB end from a different DSB induced by the same primary particle, (c) it may be joined with a DSB end from a DSB induced by a different primary particle, and (d) it may be left without being joined with any DSB end.

A DSB is assumed to be formed if two SSBs are produced on opposite strands within a certain distance, whose typical value is 10 bp. A cluster was assumed to be formed if there are two or more DSBs within a certain distance, whose typical distance is 25 bp. Therefore if there are two or more DSBs within 10 bp, over kill effect of high LET radiation should be considered. If there are two or more DSBs between 10 bp and 25 bp, effect of clustered DNA damage should be considered. If the distance between two DSBs is longer than 25 bp, they could be considered as two simple DSBs. Same as dose response of radiation-induced DSBs, the relationship between radiation dose and number of DNA fragments shorter than 30 bp induced by radiation is linear as well^35,36^. It indicates that both the clustered DNA damage effect and the over kill effect of high LET radiation depend mainly on the DSB distribution on the track of the primary particles other than the interaction among DSBs induced by different primary particles. Therefore both of the effects depend mainly on the average number of DSBs yield by each primary particle that caused DSB, that is, *λ*_*p*_ according to equation (6).

In order to be repaired correctly by NHEJ pathway, each DSB end should:

(a) not be joined with a DSB end from a DSB induced by a different primary particle. By assuming that the primary particles distribute randomly, the probability that a DSB end do not be joined with a DSB end from a DSB induced by a different primary particle^30^ is given by:

7 $${P}_{interaction}=\frac{1-{e}^{-\eta ({\lambda }_{p}){n}_{p}}}{\eta ({\lambda }_{p}){n}_{p}}$$

where *η*(*λ*_*p*_)*n*_*p*_, the average probability of a DSB end being joined with a DSB end from a DSB induced by a different primary particle, is proportional to the average number of primary particles which caused DSB, *n*_*p*_, and related to the average number of DSBs yielded by each primary particle that cause DSB, *λ*_*p*_. The relationship between *η*(*λ*_*p*_) and *λ*_*p*_ is assumed as:

8 $$\{\begin{array}{c}\eta ({\lambda }_{p})={{\rm{\eta }}}_{{\lambda }_{p}\to \infty }-\frac{{{\rm{\eta }}}_{{\lambda }_{p}\to \infty }-{\eta }_{{\lambda }_{p}\to 1}}{{\lambda }_{p}}\\ {\mathrm{lim}}_{{{\rm{\eta }}}_{{\lambda }_{p}\to 1}}\,\eta ({\lambda }_{p})={{\rm{\eta }}}_{{\lambda }_{p}\to 1}\\ {\mathrm{lim}}_{{{\rm{\eta }}}_{{\lambda }_{p}\to \infty }}\,\eta ({\lambda }_{p})={{\rm{\eta }}}_{{\lambda }_{p}\to \infty }\end{array}$$

Equation (7) quantitatively describes the interaction of DSBs induced by different primary particles.

(b) not be joined with a DSB end from a different DSB induced by the same primary particle. By assuming that a primary particle generates DSBs randomly on its track, the probability is given by:

9 $${P}_{track}=\frac{1-{e}^{-\xi {\lambda }_{p}}}{\xi {\lambda }_{p}}$$

where ξ*λ*_*p*_, the average probability of a DSB end being joined with a DSB end from a different DSB induced by the same primary particle, is proportional to *λ*_*p*_.

Equation (9) quantitatively describes the effect of clustered DNA damage.

(c) be joined with the other end from the same DSB correctly. The average probability is assumed to be *μ*_*x*_. It quantitatively describes the fidelity of NHEJ pathway.

To sum up, the probability of a DSB being correctly repaired is:

10 $${{\rm{P}}}_{correct}={\mu }_{x}{P}_{interaction}{P}_{track}={\mu }_{x}(\frac{1-{e}^{-\xi {\lambda }_{p}}}{\xi {\lambda }_{p}})(\frac{1-{e}^{-\eta ({\lambda }_{p}){n}_{p}}}{\eta ({\lambda }_{p}){n}_{p}})$$

Considering over kill effect, not all of the DSBs induced by radiation have made contribution to cell death. Similar with the effect of clustered DNA damage from equation (9), the probability of a DSB having made contribution to cell death is given by:

11 $${P}_{contribution}=\frac{1-{e}^{-\zeta {\lambda }_{p}}}{\zeta {\lambda }_{p}}$$

The sensitivity of an error repair is assumed to be *μ*_*y*_. Therefore the average number of lethal event, *N*_*death*_, can be calculated as:

12 $${N}_{death}={\mu }_{y}N\times {P}_{contribution}\times 1-{{\rm{P}}}_{correct}$$

that is,

13 $${N}_{death}={\mu }_{y}N\times (\frac{1-{e}^{-\zeta {\lambda }_{p}}}{\zeta {\lambda }_{p}})\times (1-{\mu }_{x}(\frac{1-{e}^{-\xi {\lambda }_{p}}}{\xi {\lambda }_{p}})(\frac{1-{e}^{-\eta ({\lambda }_{p}){n}_{p}}}{\eta ({\lambda }_{p}){n}_{p}}))$$

### Cell survival curve

The number of lethal event in a cell nucleus is assumed to be Poisson-distributed with an average number *N*_*death*_, therefore the cell survival *S* can be calculated as:

14 $$-lnS={N}_{death}$$

that is,

15 $$S=\exp (\,-\,{\mu }_{y}{\rm{N}}\times (\frac{1-{e}^{-\zeta {\lambda }_{p}}}{\zeta {\lambda }_{p}})\times (1-{\mu }_{x}(\frac{1-{e}^{-\xi {\lambda }_{p}}}{\xi {\lambda }_{p}})(\frac{1-{e}^{-\eta ({\lambda }_{p}){n}_{p}}}{\eta ({\lambda }_{p}){n}_{p}})))$$

Equation (7) could be Taylor expanded as:

16 $${P}_{interaction}=\frac{1-{e}^{-\eta ({\lambda }_{p}){n}_{p}}}{\eta ({\lambda }_{p}){n}_{p}}=1-\frac{1}{2}\eta ({\lambda }_{p}){n}_{p}+O(\eta ({\lambda }_{p}){n}_{p})$$

When the number of primary particles which caused DSB, *n*_*p*_, is small enough, the remainder term $O(\eta ({\lambda }_{p}){n}_{p})$ could be omitted. Therefore by substituting equation (1) and equation (16) into equation (15), the cell survival curve with the same form as the LQ model could be derived.

17 $$-lnS=\alpha D+\beta {D}^{2}$$

where

18 $$\alpha ={\rm{Y}}\times (\frac{1-{{\rm{e}}}^{-{{\rm{\zeta }}{\rm{\lambda }}}_{{\rm{p}}}}}{{{\rm{\zeta }}{\rm{\lambda }}}_{{\rm{p}}}})\times (1-{{\rm{\mu }}}_{{\rm{x}}}(\frac{1-{{\rm{e}}}^{-{{\rm{\xi }}{\rm{\lambda }}}_{{\rm{p}}}}}{{{\rm{\xi }}{\rm{\lambda }}}_{{\rm{p}}}}))\times {{\rm{\mu }}}_{y}$$

19 $${\rm{\beta }}=\frac{1}{2}{\rm{\eta }}({{\rm{\lambda }}}_{{\rm{p}}})\frac{{\rm{Y}}}{{{\rm{\lambda }}}_{{\rm{p}}}}\times {\rm{Y}}\times (\frac{1-{{\rm{e}}}^{-{{\rm{\zeta }}{\rm{\lambda }}}_{{\rm{p}}}}}{{{\rm{\zeta }}{\rm{\lambda }}}_{{\rm{p}}}})\times (\frac{1-{{\rm{e}}}^{-{{\rm{\xi }}{\rm{\lambda }}}_{{\rm{p}}}}}{{{\rm{\xi }}{\rm{\lambda }}}_{{\rm{p}}}})\times {{\rm{\mu }}}_{{\rm{x}}}{{\rm{\mu }}}_{{\rm{y}}}$$

It shows that for a specific cell, one or more DSBs induced by single primary particle and their interactions, including the DNA clustered damage effect and the over kill effect, contribute to α term of the LQ model. The interaction among DSBs induced by different primary particles, which depends mainly on the number of primary particles causing DSB, contributes to β term of the LQ model.

## Results

### Yield of radiation-induced DSB

**Figure 1:**
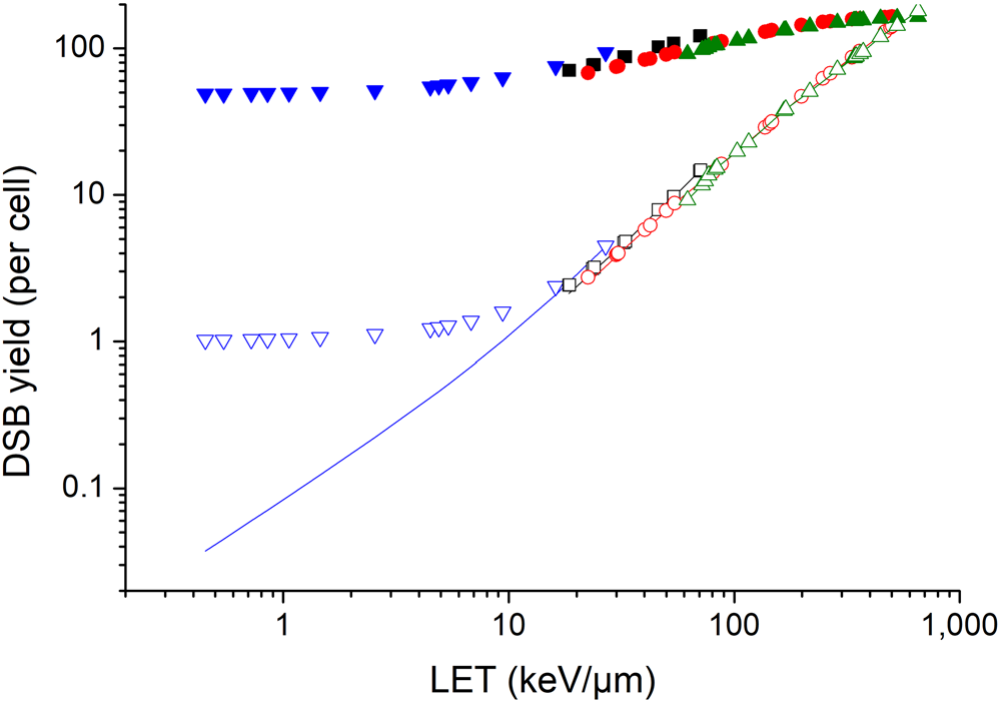
Yield of radiation-induced DSB of human cells. Solid points represent DSB yield per Gy, Y, of human cells irradiated with proton(), He-3 ion (■), C-12 ion () and Ne-20 ion (). Hollow points with the same color represent DSB yield per track, λ. Solid lines with the same color represent the number of DSBs yielded by each primary particle that causes DSB, *λ*_*p*_.

The experimental data of cell survival curves were from the PIDE database^37^, in which the published results of *in-vitro* cell survival experiments were collected. In this study, experimental data of HSG cell and V79 cell were used since there are many published studies based on HSG cell and V79 cell. The content of DNA in the human nucleus was set to 6 Gbp, and the content of DNA in the nucleus of Chinese hamster was set to 5.6 Gbp in the calculations with MCDS. Figure 1 shows the results of human cells exposed to proton, He-3 ion, C-12 ion, and Ne-20 ion. It shows that both the DSB yield per Gy, *Y*, and the DSB yield per track, *λ*, increase with LET. According to equation (2), when the dose is constant, the number of primary particles passing through the cell nucleus is inversely proportional to LET, therefore, DSB yield per track changes much more quickly with LET.

With the *Y* and *λ* obtained with MCDS, *n*_*p*_ and *λ*_*p*_ were calculated with equation (5) and equation (6). Figure 1 shows the difference between *λ* and *λ*_*p*_ For proton at LET below 10 keV/μm, the DSB yield per track increases quickly with LET, however, the average number of DSBs induced by each primary particle that causes DSB increases quite slowly and it is slightly higher than one, which is similar with photons. It may be an explanation why biological effectiveness of low LET proton is similar with or slightly higher than photons. The difference between *λ* and *λ*_*p*_ is negligible for high-LET radiations.

### Model parameter fitting

The parameters of the model were obtained by fitting the experimental data of survival curves of HSG cell (54 cell survival curves) and V79 cell (52 cell survival curves) from Furusawa *et al*. in 2000^38^. Table 1 shows the parameters of the model for HSG cell and V79 cell.

**Table 1:** Parameters of the cell survival model following radiation-induced DSBs for HSG cell and V79 cell.

Parameter	HSG	V79
*μ*_*x*_	0.9817 ± 0.0056	0.9568 ± 0.0236
*μ*_*y*_	0.0891 ± 0.0068	0.0300 ± 0.0177
ζ	0.1025 ± 0.0065	0.0412 ± 0.0209
ξ	0.0572 ± 0.0027	0.0608 ± 0.0381
η*λ*_*p*_→1	(7.26 ± 0.04)E-4	(9.78 ± 0.10)E-4
η*λ*_*p*_→∞	0.0022 ± 0.0001	0.0065 ± 0.0001

The values are presented as best fit parameters ± one standard deviation fitting uncertainty.

**Figure 2:**
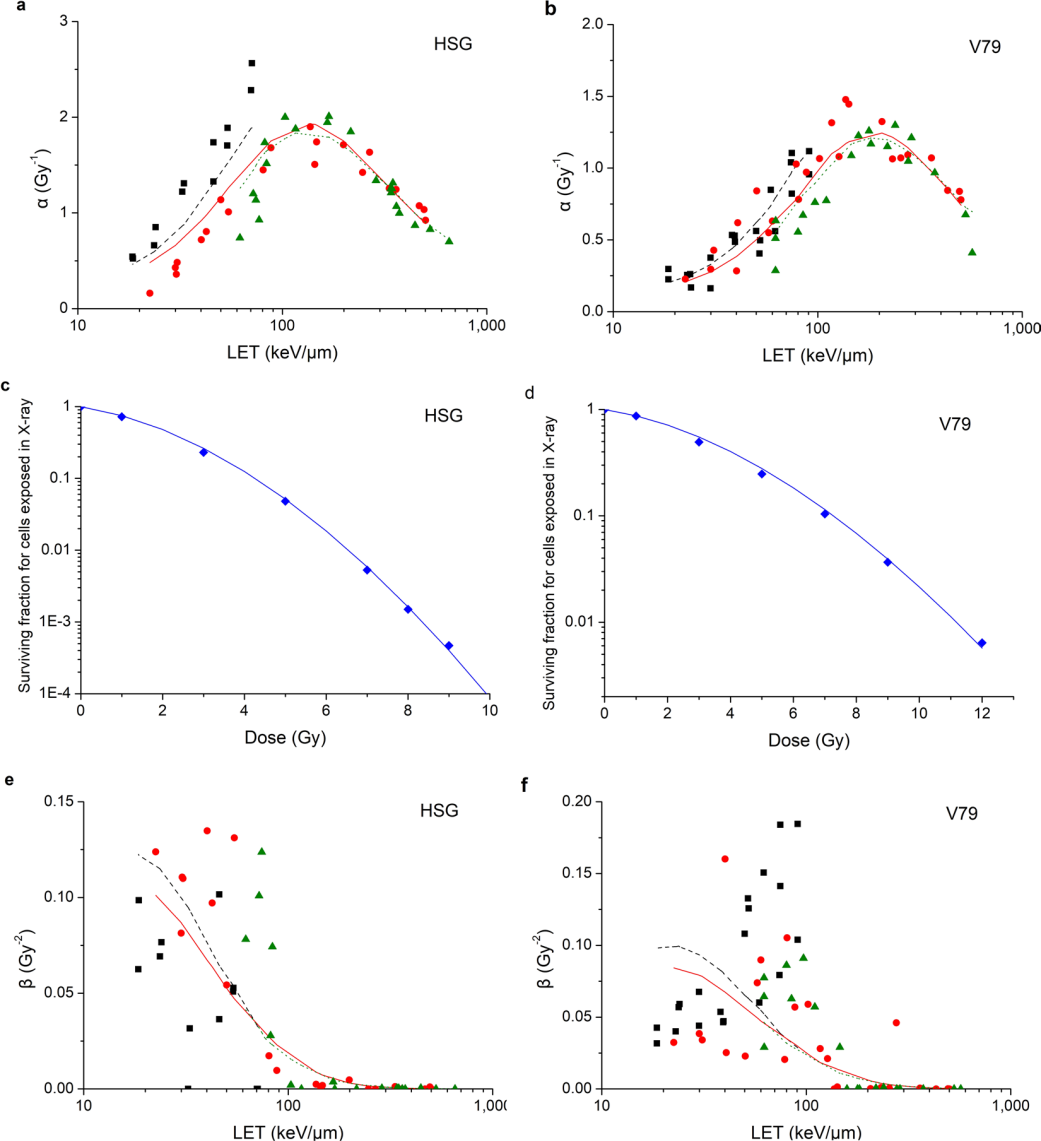
Comparison between observed values from cell survival experiments (■ He-3 ion,  C-12 ion,  Ne-20 ion,  X-ray) and modelled values in this study (line with the same color as points) for HSG cell and V79 cell. (**a**) is the comparison between observed α values and modelled α values for HSG cell and (**b**) is for V79 cell. (**c**) is the survival curve for HSG cell exposed in X-ray and (**d**) is for V79 cell exposed in X-ray. (**e**) is the comparison between observed β values and modelled β values for HSG cell and (**f**) is for V79 cell.

Firstly *μ*_*x*_, *μ*_*y*_, ζ and ξ were obtained with the experimental data of α values as well as the calculated *n*_*p*_ and *λ*_*p*_ Consequently the modelled data of α values could be obtained with equation (18). Comparison between observed α values from cell survival experiments and modelled α values in this study is shown in Fig. 2a,b. It indicates that the modelled α values are in good agreement with the observed α values. The correlation coefficient is R^2^ = 0.7755 for HSG cell while R^2^ = 0.8522 for V79 cell.

Then, with the *μ*_*x*_, *μ*_*y*_, ζ and ξ obtained above as well as the experimental data of cell survival in X-ray, η_*λp*→1_ was obtained with equation (15). Consequently, the modelled data of surviving fractions could also be obtained. The survival curves for HSG cell and V79 cell exposed in X-ray are shown in Fig. 2c,d. It indicates that the modelled data agree well with the experimental data. The correlation coefficient is R^2^ = 0.9991 for HSG cell while R^2^ = 0.9986 for V79 cell.

Finally with the *μ*_*x*_, *μ*_*y*_, ζ, ξ and η_*λp*→1_ obtained above, η_*λp*→∞_ was obtained with the experimental data of β values. Consequently, the modelled data of β values could be obtained with equation (19). Comparison between observed β values from cell survival experiments and modelled β values in this study is shown in Fig. 2e,f. It indicates that the variation trend of modelled and observed β values with LET are in agreement. The correlation coefficient is R^2^ = 0.2008 for HSG cell while R^2^ = 0.1477 for V79 cell. The correlation coefficients are small and it may be attributed to two factors. One factor is that the experimental data of β values are relatively scattered for radiation at LET less than 100 keV/μm. The other factor is that the difference between α values and β values is about 2 orders of magnitude for radiation at LET larger than 100 keV/μm, therefore, the contribution of β values to cell survival could hardly be shown in experimental data.

### Surviving Fraction calculation with the model

**Figure 3:**
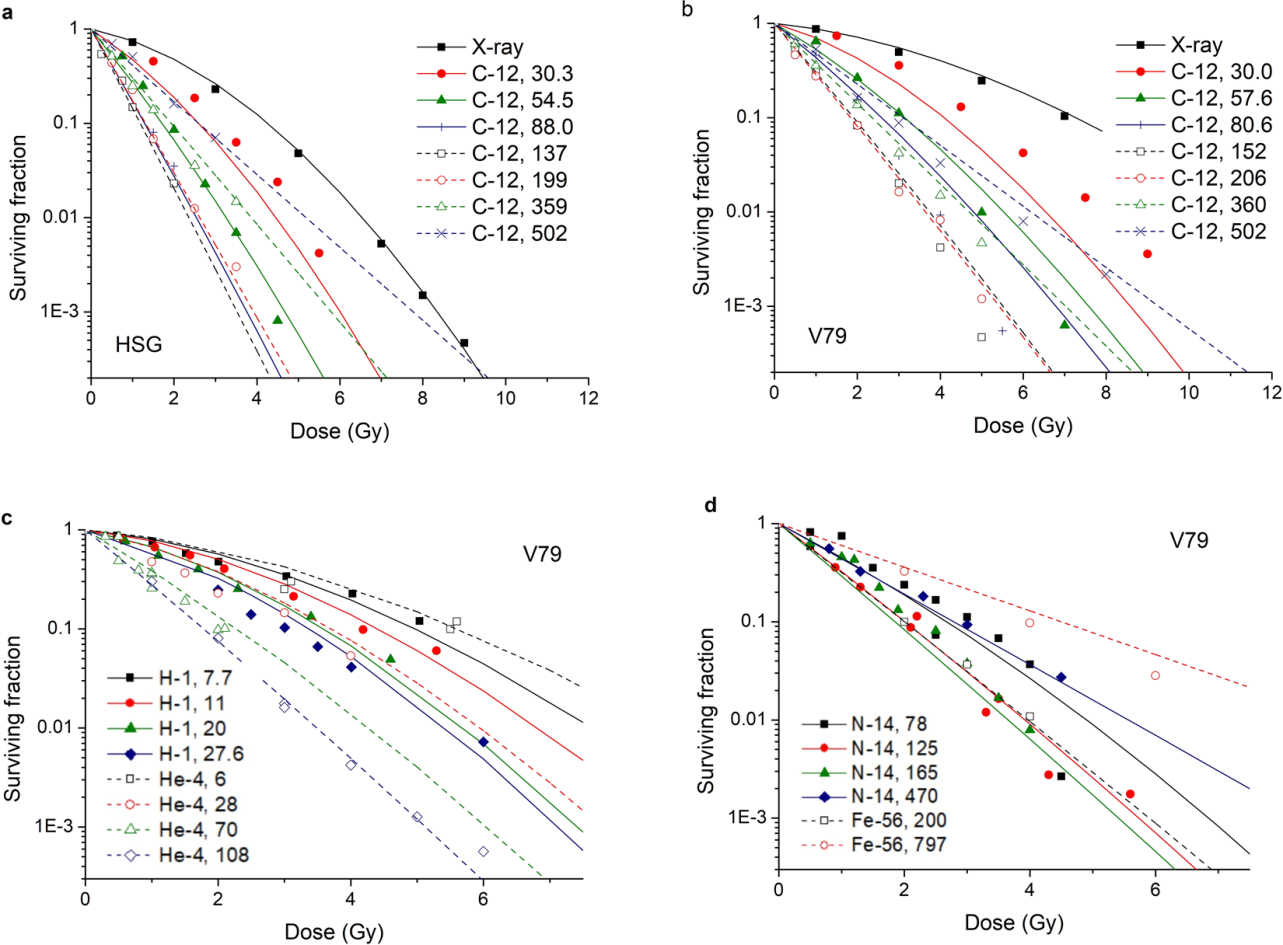
Comparison between experimental data of cell surviving fractions (points) and modelled data of cell surviving fractions in this study (lines). (**a**) is the comparison between experimental data and modelled data for HSG cell irradiated by C-12 ion at different LET and X-ray. (**b**) is for V79 cell irradiated by C-12 ion at different LET and X-ray. (**c**) is for V79 cell irradiated by H-1 ion^41,42^ and He-4 ion^43–45^. (**d**) is for V79 cell irradiated by N-14 ion^43,44,46^ and Fe-56 ion^47^.

With the model and the parameters obtained above, cell survival for HSG cell and V79 cell irradiated by C-12 ion at different LET could be calculated. Comparison between experimental data of cell survival and the modelled values in this study is shown in Fig. 3a,b. It shows that the modelled values are in good agreement with the experimental data not only at low LET, but also at high LET. The correlation coefficient is R^2^ = 0.9808 for HSG cell while R^2^ = 0.9192 for V79 cell.

For the same cell type, the model is able to calculate the surviving fraction not only for the particle types used in model parameter fitting, but also for different types of particles at different LET. Taking V79 cells as an example, the comparison between other experimental data of different particles at different LET and the modelled surviving fractions was shown in Fig. 3c,d. It can be seen that the modelled values are in good agreement with the experimental values.

### RBE calculation with the model

In recent years, advanced radiotherapy techniques which make use of protons and carbon ions have been widely applied. Besides the advantage of a sharp Bragg peak with a steep dose falloff downstream, there is an enhanced RBE for heavy charged particles. RBE depends not only on the physical parameters of the irradiation but also on the biological parameters of the irradiated biological system^12^, which presents the challenge of estimating the RBE precisely. The model is able to calculate RBE for the same type of irradiated cells. It allows not only to calculate RBE at different cell survivals, but also to predict RBE for different types of particles at different LET.

**Figure 4:**
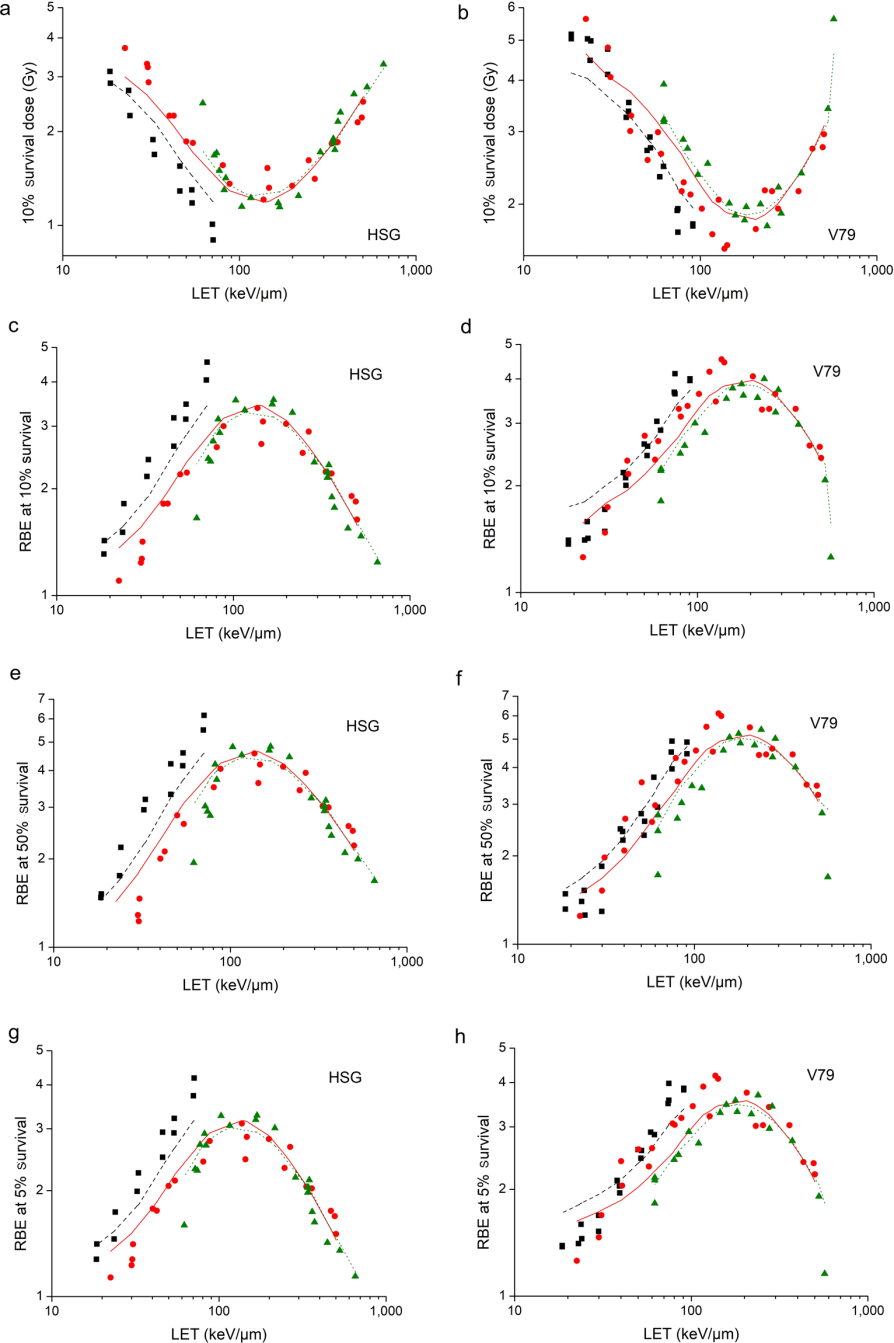
Comparison between observed RBE values from cell survival experiments (■ He-3 ion,  C-12 ion,  Ne-20 ion) and modelled values in this study (line with the same color as points) for HSG cell and V79 cell. (**a**) is the comparison between observed values of 10% survival dose and modelled values for HSG cell and (**b**) is for V79 cell. (**c–h**) are comparisons between observed RBE values at 10%, 50% and 5% surviving fractions and modelled values for HSG cell and V79 cell.

With the model parameters obtained in Table 1, the modelled data of 10% survival dose were calculated. Comparison between observed values from cell survival experiments and modelled values is shown in Fig. 4a,b. It shows that the modelled values are in good agreement with observed values. The correlation coefficient is R^2^ = 0.8608 for HSG cell while R^2^ = 0.8914 for V79 cell. Consequently, the modelled data of RBE at 10% survival were calculated. Comparison between observed values from cell survival experiments and modelled values is shown in Fig. 4c,d. It shows that the modelled values are also in good agreement with observed values. The correlation coefficient is R^2^ = 0.8291 for HSG cell while R^2^ = 0.8785 for V79 cell.

According to Fig. 2, the modelled α and β values are in good agreement with the experimental data. According to Fig. 3, the modelled surviving fractions are in good agreement with the experimental data as well. Therefore, the model is able to calculate RBE at a different survival. Comparison between observed values of RBE at 50% survival and RBE at 5% survival from cell survival experiments and modelled values is shown in Fig. 4e–h. It shows that the modelled values are in good agreement with observed values. The correlation coefficient of RBE at 50% survival and RBE at 5% survival for HSG cell is R^2^ = 0.7997 and R^2^ = 0.8306 respectively. The correlation coefficient of RBE at 50% survival and RBE at 5% survival for V79 cell is R^2^ = 0.8735 and R^2^ = 0.8491 respectively.

**Figure 5:**
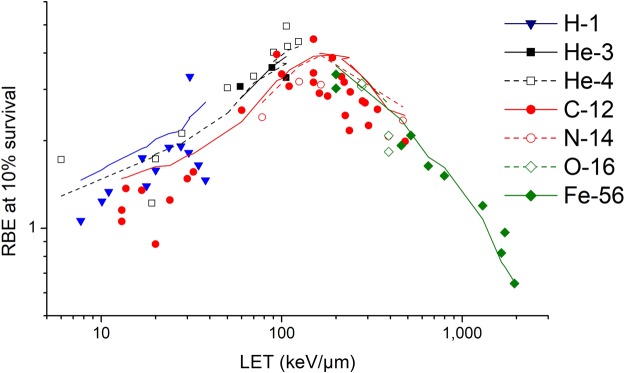
Comparison of RBE at 10% survival for V79 cells irradiated by charged particles. The points are observed RBE for H-1^41,42,45^, He-3^41^, He-4^43–45,48–51^, C-12^39,47,49,52–57^, N-14^43,44,46^, O-16^55^ and Fe-56^47,55^ ion, and the lines with the same color are modelled values.

The model is also able to calculate the RBE values for the same type of cells irradiated by different particles at different LET. Taking V79 cells as an example, the comparison of the modelled RBE values at 10% survival and the RBE values from other experimental data is shown in Fig. 5. The experimental data of cell survival curves for V79 cells irradiated by charged particles were from the PIDE database^37^.The correlation coefficient is R^2^ = 0.7620. It can be seen that the trend of modelled RBE changing with LET is good agreement with the trend of experimental data for the same type of cell irradiated by different particles at different LET.

### Biological parameters of different cell types

The model is able to reflect the biological parameters of the irradiated cells, such as the α/β ratio, the cluster DNA damage effect and the over kill effect. The ratio α/β in the LQ model was regarded as an indicator of cellular repair capacity by some researchers^39,40^. According to equation (18) and equation (19), the α/β ratio can be calculated with:

20 $$\alpha /{\rm{\beta }}=\frac{{\rm{Y}}\times (\frac{1-{{\rm{e}}}^{-{{\rm{\zeta }}{\rm{\lambda }}}_{{\rm{p}}}}}{{{\rm{\zeta }}{\rm{\lambda }}}_{{\rm{p}}}})\times (1-{{\rm{\mu }}}_{{\rm{x}}}(\frac{1-{{\rm{e}}}^{-{{\rm{\xi }}{\rm{\lambda }}}_{{\rm{p}}}}}{{{\rm{\xi }}{\rm{\lambda }}}_{{\rm{p}}}}))\times {{\rm{\mu }}}_{y}\,}{\frac{1}{2}{\rm{\eta }}({{\rm{\lambda }}}_{{\rm{p}}})\frac{{\rm{Y}}}{{{\rm{\lambda }}}_{{\rm{p}}}}\times {\rm{Y}}\times (\frac{1-{{\rm{e}}}^{-{{\rm{\zeta }}{\rm{\lambda }}}_{{\rm{p}}}}}{{{\rm{\zeta }}{\rm{\lambda }}}_{{\rm{p}}}})\times (\frac{1-{{\rm{e}}}^{-{{\rm{\xi }}{\rm{\lambda }}}_{{\rm{p}}}}}{{{\rm{\xi }}{\rm{\lambda }}}_{{\rm{p}}}})\times {{\rm{\mu }}}_{{\rm{x}}}{{\rm{\mu }}}_{{\rm{y}}}\,}=\frac{1-{{\rm{\mu }}}_{{\rm{x}}}(\frac{1-{{\rm{e}}}^{-{{\rm{\xi }}{\rm{\lambda }}}_{{\rm{p}}}}}{{{\rm{\xi }}{\rm{\lambda }}}_{{\rm{p}}}})}{\frac{1}{2}{{\rm{\mu }}}_{{\rm{x}}}{\rm{\eta }}({{\rm{\lambda }}}_{{\rm{p}}})\frac{{\rm{Y}}}{{{\rm{\lambda }}}_{{\rm{p}}}}}$$

For photon and low-LET irradiation, *λ*p→1 the α/β ratio is mainly attributed to the fidelity of NHEJ pathway and the interaction of DSBs induced by different primary particles. However, for charged particles at higher LET, the α/β ratio is mainly attributed to *Y/λp*, the number of primary particles which caused DSB per unit dose. It may be an explanation why cell sensitivity has less effect on cell killing for charged particles than photons.

**Figure 6:**
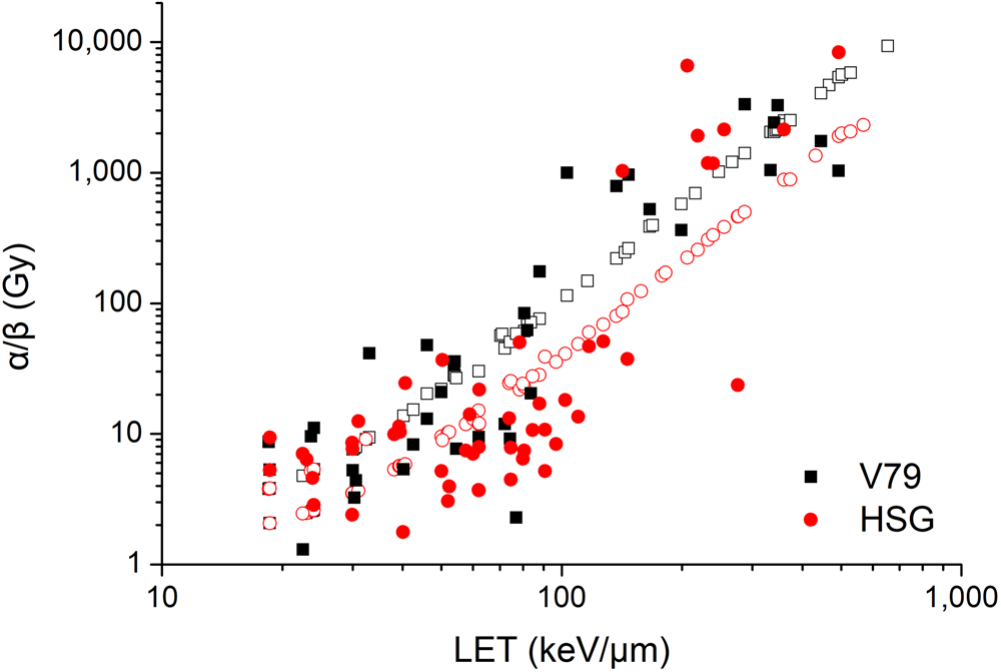
Comparison of experimental data of α/β ratio for HSG cells () and V79 cells (■) and modelled results (open points with the same color).

Figure 6 shows the α/β ratio for HSG cells and V79 cells irradiated by different charged particles at different LET. The solid points are experimental data (β≠0) from Furusawa *et al*., and the open points are modelled results. The α/β ratio increases quickly with LET, which is mainly attributed to two factors. One factor is that the α value increases with LET because of the cluster DNA damage effect, the other factor is that the number of primary particles delivering unit dose to the nucleus decreases with LET so that the interaction of DSBs induced by different primary particles decreases. For radiation at LET larger than 100 keV/μm, the interaction among different primary particles is vanishingly small and the difference between α values and β values is above 2 orders of magnitude, therefore, the contribution of β values to cell survival could hardly be shown in experimental data.

**Figure 7:**
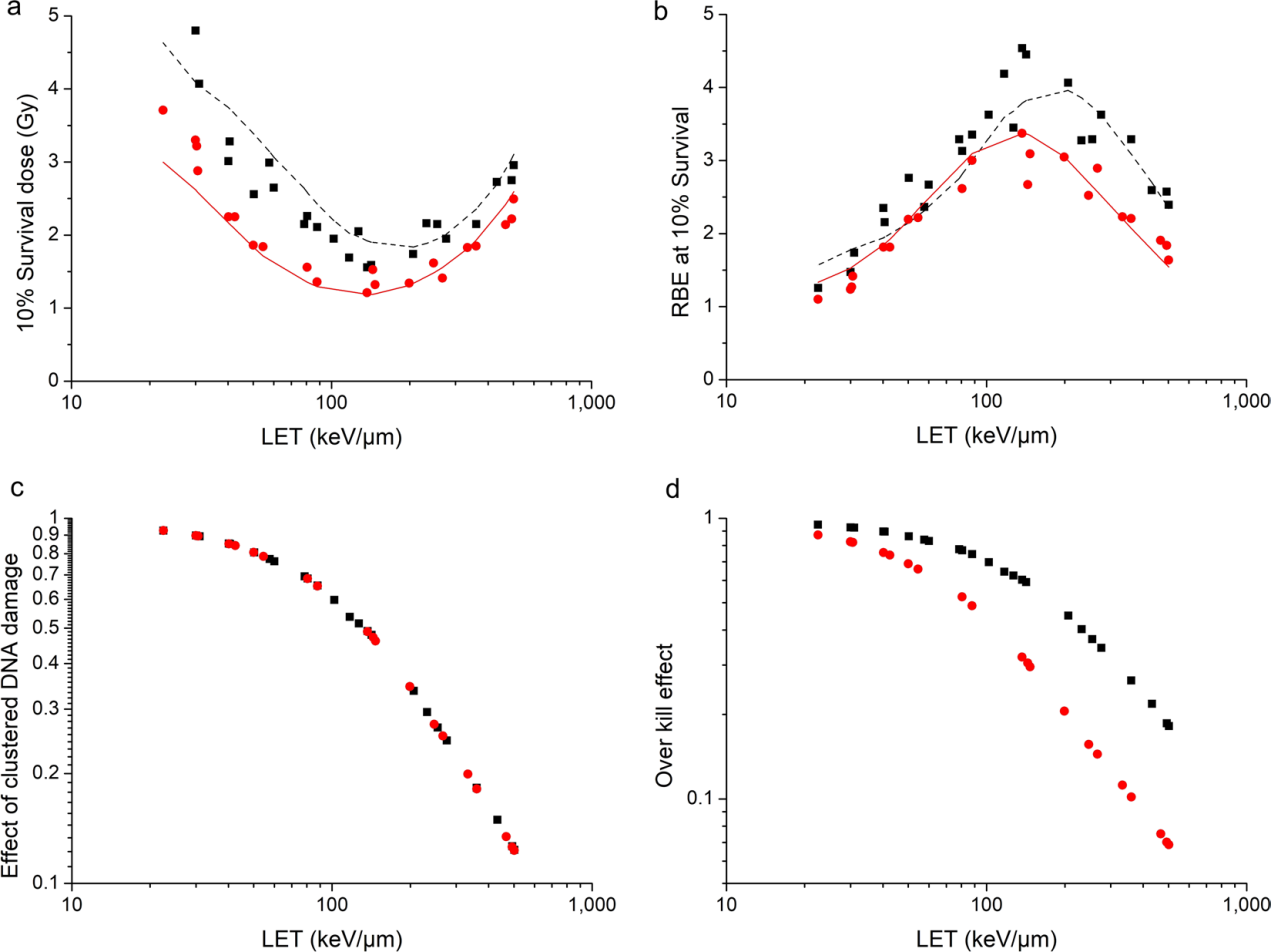
Clustered DNA damage effect as well as over kill effect on HSG cells () and V79 cells (■) irradiated by C-12 ions. (**a**) is the comparison of 10% survival dose between HSG cells and V79 cells and (**b**) is the comparison of RBE at 10% survival. The points are experimental data and the lines with the same color are modelled values. (**c**) is the comparison of effect of clustered DNA damage calculated with equation (9). (**d**) is the comparison of over kill effect calculated with equation (11).

The model is able to reflect the cluster DNA damage effect and the over kill effect on the irradiated cells as well. For HSG cells and V79 cells exposed to C-12 ions, the comparison of 10% survival dose as well as the comparison of RBE at 10% survival is shown in Fig. 7a,b. HSG cells are more sensitive than V79 cells when irradiated by X-ray.The 10% survival dose of X-ray for HSG cells is 4.08 Gy (obtained from experimental data of cell survival curve), and the 10% survival dose of X-ray for V79 cells is 7.07 Gy. When irradiated by C-12 ions with different LET, the difference between 10% survival dose for HSG cells and that for V79 cells decreases with the LET, while the difference of RBE values is subtle when LET is lower than 100 keV/μm, and the difference is relatively obvious when LET is higher than 100 keV/μm. It could be explained by the cluster DNA damage effect and the over kill effect.

Figure 7c,d are comparisons of effect of clustered DNA damage calculated with equation (9) as well as over kill effect calculated with equation (11) between HSG cells and V79 cells irradiated by C-12 ions. It can be seen that the cluster damage effect of HSG cells and V79 cells is consistent, while the over kill effect of HSG cells is more obvious than that of V79 cells. Therefore, when LET is less than 100 keV/μm, cluster DNA damage effect dominates, RBE difference between HSG cells and V79 cells is not significant. When LET is larger than 100 keV/μm, over kill effect dominates gradually, and the RBE difference between HSG cells and V79 cells becomes greater.

## Discussion

A mechanistic model of cellular survival following radiation-induced DSBs has been proposed to predict the relationship between radiation-induced DSBs in cell nucleus and probability of cell survival. It was assumed that DSBs were the initial lesions in the DNA of the nucleus induced by ionizing radiation and NHEJ was the main pathway of DSB repair in mammalian cells. The effect of DNA clustered damage and the over kill effect were considered in the model. As only NHEJ pathway is considered in the proposed model and HRR pathway is not considered, the model is suitable to cell phases when NHEJ pathway domains.

There were two input parameters in the proposed model, the average number of primary particles which caused DSB and the average number of DSBs yielded by each primary particle that caused DSB. The former was used to describe the contribution of interaction among DSBs induced by different primary particles. The latter was used to describe the contribution of DSBs induced by single primary particle and their interactions, including the cluster DNA damage effect and the over kill effect. There were six fitting parameters used to describe the biological characteristics of the irradiated cells, which could be obtained with experimental data. By determining the fitting parameters with experimental data, the model allowed to estimate surviving fractions for the same type of cell exposed to different particles at different LET, and then to estimate the RBE at different survival. The model further revealed the mechanism of cell death induced by the DSB effect, as well as reflected the biological parameters of the irradiated cells.

Firstly, the contribution of DSBs to α term and β term of the LQ model was discussed. Since dose response of DSB induced by radiation is linear, one or more DSBs induced by single primary particle and their interactions contribute to α term of the LQ model. The interaction among DSBs induced by different primary particles, which depends mainly on the number of primary particles which cause DSB, contributes to β term of the LQ model. Therefore the cell survival curve is in agreement with the LQ model for low-LET radiations. However, for high-LET radiations, when delivering the same dose to the nucleus as low-LET radiations, the number of primary particles causing DSB is much smaller, and the contribution of interaction among DSBs induced by different primary particles to cell death would be vanishingly small, therefore the cell survival curve is in agreement with the exponential model.

Secondly, the effect of clustered DNA damage and the effect of over kill on cell death were quantitatively described. Both the clustered DNA damage effect and the over kill effect depend on the average number of DSBs induced by single primary particle that causes DSB. The primary particles passing through the nucleus without causing any DSB are considered as having made no contribution to cell death. For protons at LET below 10 keV/μm, the DSB yield per track increases quickly with LET, however, the average number of DSBs induced by each primary particle that causes DSB increases quite slowly and it is slightly higher than one, which is similar with photons. It may be an explanation why biological effectiveness of low-LET protons is similar with or slightly higher than photons. In the LET range from 100 to 200 keV/μm, the contribution of the over kill effect is not obvious, and the contribution of the clustered DNA damage effect is dominated, therefore the RBE is much larger. When LET is higher than 200 keV/μm, the contribution of the over kill effect is gradually obvious, and finally exceeds the contribution of the cluster DNA damage effect, therefore the RBE decreases gradually.

Thirdly, the ratio α/β, which was regarded the as an indicator of cellular repair capacity by some researchers, was quantitatively described in the model. The α/β ratio increases quickly with LET, which is mainly attributed to two factors. One factor is that the α value increases with LET because of the cluster DNA damage effect, the other factor is that the number of primary particles delivering unit dose to the nucleus decreases with LET so that the interaction of DSBs induced by different primary particles decreases. For radiations at LET larger than 100 keV/μm, the interaction among different primary particles is vanishingly small and the difference between α values and β values is above 2 orders of magnitude, therefore the contribution of β values to cell survival could hardly be shown in experimental data.

Finally, the RBE of charged particles could be calculated with the proposed model. On the one hand, the model with parameters determined by the experimental data allowed to estimate surviving fractions of the same type of cell irradiated by different particles at different LET. Consequently it allowed to estimate the RBE of charged particles; on the other hand, because the trend of modelled α and β values changing with LET is in good agreement with the experimental data, the model applies not only to the estimation of RBE at 10% survival, but also to the estimation of RBE at a different survival such as 5%, which provides reference for larger dose segmentation in the clinical treatment.

In conclusion, we have developed a mechanistic model of cellular survival following radiation-induced DSBs to predict the relationship between radiation-induced DSBs in cell nucleus and probability of cell survival. The model further revealed the mechanism of cell death induced by the DSB effect. It has the power to reflect the biological parameters of the irradiated cells as well. Cellular survival after irradiation and RBE of charged particles could be calculated with the proposed model, which would provide reference for clinical treatment.
